# Clinical Aspects of Melatonin Intervention in Alzheimer’s Disease Progression

**DOI:** 10.2174/157015910792246209

**Published:** 2010-09

**Authors:** Daniel P Cardinali, Analía M Furio, Luis I Brusco

**Affiliations:** 1 Departamento de Docencia e Investigación, Facultad de Ciencias Médicas, Pontificia Universidad Católica Argentina, Buenos Aires, Argentina; 2 Departamento de Fisiología, Facultad de Medicina, Universidad de Buenos Aires, Buenos Aires, Argentina; 3 Hospital de Clínicas “José de San Martín”, Universidad de Buenos Aires, Buenos Aires, Argentina

**Keywords:** Melatonin, Alzheimer's disease, minimal cognitive impairment, neuropsychological tests, clinical trials.

## Abstract

Melatonin secretion decreases in Alzheimer´s disease (AD) and this decrease has been postulated as responsible for the circadian disorganization, decrease in sleep efficiency and impaired cognitive function seen in those patients. Half of severely ill AD patients develop chronobiological day-night rhythm disturbances like an agitated behavior during the evening hours (so-called “sundowning”). Melatonin replacement has been shown effective to treat sundowning and other sleep wake disorders in AD patients. The antioxidant, mitochondrial and antiamyloidogenic effects of melatonin indicate its potentiality to interfere with the onset of the disease. This is of particularly importance in mild cognitive impairment (MCI), an etiologically heterogeneous syndrome that precedes dementia. The aim of this manuscript was to assess published evidence of the efficacy of melatonin to treat AD and MCI patients. PubMed was searched using Entrez for articles including clinical trials and published up to 15 January 2010. Search terms were “Alzheimer” and “melatonin”. Full publications were obtained and references were checked for additional material where appropriate. Only clinical studies with empirical treatment data were reviewed. The analysis of published evidence made it possible to postulate melatonin as a useful ad-on therapeutic tool in MCI. In the case of AD, larger randomized controlled trials are necessary to yield evidence of effectiveness (i.e. clinical and subjective relevance) before melatonin´s use can be advocated.

## INTRODUCTION

Oxidative damage has been suggested as the primary cause of aging and age-associated neurodegenerative diseases like Alzheimer’s disease (AD). This concept is based on the free radical hypothesis of aging as proposed by Harman more than 50 years ago [27]. Many recent reviews on AD present compelling evidence for a decisive participation of severe oxidative stress in the development of neuropathology seen in this disease [1, 5, 6, 10, 19, 57, 65, 71, 73, 74]. Therefore, numerous compounds with antioxidant properties have been suggested for treatment of AD and other neurodegenerative diseases [24, 42, 61, 62, 78, 81]. 

Among these substances, melatonin emerges as unique for several reasons: it is a natural compound synthesized in the pineal gland and other body tissues [63], it can be released by the pineal gland *via *the pineal recess into the cerebrospinal fluid (CSF), in much higher concentrations than into the circulation [33, 76], and its production decreases with age, a fact which has been suggested to a the major predisposing factor in neurodegenerative diseases [52, 71]. This review focuses on the therapeutic potential of melatonin in AD and in minimal cognitive impairment (MCI), an etiologically heterogeneous syndrome which progresses to AD at an approximate rate of 12 % every year. Since MCI can represent prodromal AD it needs to be adequately diagnosed and treated [16]. 

## MELATONIN AND ALZHEIMER’S DISEASE

AD is an age-associated neurodegenerative disease that is characterized by a progressive loss of cognitive function, loss of memory, and other neurobehavioral manifestations. In spite of a large number of studies undertaken, the etiology of AD remains largely unknown, although a participation of oxidative stress in the development of neuropathology seems to be warranted. Many mechanisms have been proposed as predisposing for excessive oxidative damage, including the genetic background (e.g., expression levels and subforms of presenilins and apolipoprotein E), inflammatory processes associated with cytokine release, or neurotoxicity by metal ions [1, 5, 6, 10, 19, 57, 65, 71, 73, 74]. The deposition of β-amyloid (Aβ) plaques is thought to destabilize neurons by mechanisms which require further clarification. Tangles are associated with hyperphosphorylation of tau, a microtubule-associated protein, and of neurofilament H/M subunits, processes that lead to misfolding and accumulation of altered proteins, along with a disruption of microtubules [4, 7, 31, 32, 55, 60].

However, the simplistic concept that reduces AD lesions to oxidative damage has been shown insufficient to explain the disease [24, 42, 61, 62, 78, 81]. Classical radical scavengers like vitamins E and C have been used for the treatment of AD patients with only limited success. Although some studies demonstrated a reduction in lipid peroxidation [30], epidemiological data showed only minor or no clear-cut clinical effects of classical antioxidants [24, 42, 61, 62, 78, 81]. Moreover, these compounds remained relatively inefficient in preventing Aβ toxicity and fibrillogenesis [31, 35, 72]. 

In this regard, melatonin and other structurally related indolic compounds, such as indole-3-propionic acid, proved to be more potent than classical antioxidants [12, 13, 53, 58]. The antifibrillogenic effects of melatonin and its metabolites were observed not only *in vitro* but also *in vivo* in transgenic mouse models [18, 40, 49]. Protection from Aβ toxicity was observed, especially at the mitochondrial level. In addition melatonin and its related compounds display particular chronobiological properties that make them capable of correcting the circadian rhythm disorders seen in AD patients. 

Many of these actions of melatonin were demonstrated at elevated, pharmacological concentrations, but any judgment of the physiological relevance of such findings has to consider the relatively high rates of melatonin secretion into the CSF, uptake into the brain tissue and, presumably also, the metabolism to other protective compounds, such as the *N*^1^-acetyl-*N*^2^-formyl-5-methoxykynuramine and *N*^1^-acetyl-5-methoxykynuramine [26], processes which are impaired during aging and in neurodegenerative diseases.

## MELATONIN LEVELS IN ALZHEIMER’S DISEASE

Several studies have showed that melatonin levels are diminished in AD patients compared to age-matched control subjects [34, 39, 46, 48, 70, 77]. CSF melatonin levels decrease even in preclinical stages when the patients do not manifest any cognitive impairment (at Braak stages I-II), suggesting that the reduction in melatonin is an early marker for the first stages of AD [82, 84]. 

The reduction in nocturnal melatonin levels with the abolition of diurnal melatonin rhythmicity can be the consequence of dysfunction of noradrenergic regulation and depletion of the melatonin precursor serotonin as already seen in the earliest preclinical AD stages [82]. Alternatively, changes in the pathways of light transmission, from physical properties of the dioptric apparatus to a defective retinohypothalamic tract or suprachiasmatic nuclei (SCN)-pineal connections have been discussed as possible reasons for the decline in amplitude of the melatonin rhythm and the corresponding changes in the circadian system [69]. One should, however, be aware that light is inhibitory to the pineal, so that dysfunction in the transmission of light signals would not easily explain a decrease in melatonin. In any case, it is clear that the changes in melatonin secretion contribute to the frequent symptoms of sleep disruption, nightly restlessness and sundowning seen in AD patients.

Other reasons may be sought in an altered metabolism of AD patients, e.g., in relation to known genetic predispositions. The presence of apolipoprotein E-ε4/4, which is associated with enhanced Aβ toxicity and more rapid disease progression, also leads to considerably stronger declines in melatonin in the respective AD subpopulation than in patients with other apolipoprotein subtypes [34]. From this point of view, the relative melatonin deficiency appears as a consequence rather than as one of the causes of AD, although the loss in melatonin could aggravate the disease. Decreased nocturnal melatonin levels were shown to correlate with the severity of mental impairment of demented patients [36].

## SLEEP-WAKE AND CIRCADIAN RHYTHM ABNORMALITIES IN AD PATIENTS

The sleep-wake disturbances in AD patients become more marked with progression of the disease. Sleep-wake disturbances of elderly AD patients result from changes at different levels, such as reductions in the strength of environmental synchronizers or their perception, a lack of mental and physical activity, age- or disease-induced losses of functionality of the circadian clock. Cross-sectional studies have shown that sleep disturbances are associated with increased memory and cognitive impairment in AD patients [41]. 

AD patients with disturbed sleep-wake rhythms did not only exhibit reduced amounts of melatonin secreted, but also a higher degree of irregularities in the pattern of the melatonin rhythm, such as variations in phasing of the peak [46]. Therefore, the melatonin rhythm has not only lost signal strength in clock resetting, but also reliability as an internal synchronizing time cue. Loss or damage of neurons in the hypothalamic SCN and other parts of the circadian timing system presumably account for the circadian rhythm abnormalities seen in demented patients [50, 51, 79], especially as the number of neurons in the SCN of AD patients is reduced [43]. Clinical findings strongly argue in favor of disruption of the circadian timing system in AD, since numerous overt rhythms are disturbed, including body temperature and concentrations of other hormones such as glucocorticoids [25, 28]. Circadian alterations, which are detectable at an advanced stage of AD, also concern phase relationships, such as the phase difference between the rest-activity and core body temperature cycles, the last one being significantly delayed [79]. 

In facing a weakened circadian system the possibility of improving rhythmicity in AD patients by well-timed light treatment has been entertained [66]. In practical terms, this may be important as AD patients were found to be less exposed to environmental light than their age-matched controls, so that dysfunction of the SCN was aggravated by low strength of the synchronizing signal light. There is evidence that the combined treatment of bright light plus melatonin showed the best effects to attenuate cognitive deterioration and to improve sleep in old patients [15, 64]. In other words, the AD patient is gradually deprived of the photic input and even more of the non-photic, darkness-related internal signal melatonin.

Sundowning, the typical chronobiological phenomenon seen in AD, is observed in conjunction with disturbances of the sleep-wake cycle. Symptoms appeared in the late afternoon or early evening and include reduced ability to maintain attention to external stimuli, disorganized thinking and speech, a variety of motor disturbances including agitation, wandering and repetitious physical behaviors and perceptual and emotional disturbances [66, 75]. Bright light exposure in selected circadian phases markedly alleviated sundowning symptoms, such as wandering, agitation and delirium and improved sleep wave patterns in AD patients [44, 83]. Therefore, it was logical to test whether melatonin could be an effective resynchronizing agent (or chronobiotic) to improve this AD patient's condition.

## MELATONIN AS A THERAPEUTIC AGENT FOR ALZHEIMER’S DISEASE

Melatonin (3 mg p.o. for 21 days) as a sleep-promoting agent was first tried in a small non-homogenous group of elderly patients with primary insomnia associated with dementia [17]. Seven out of 10 dementia patients having sleep disorders treated with melatonin (3 mg p.o. at bed time) showed a significant decrease in sundowning and reduced variability of sleep onset time. In another study, 14 AD patients who exhibited irregular sleep-wake cycles, treated with 6 mg for 4 weeks, showed a significantly reduced percentage of nighttime activity as compared to a placebo group [45]. The efficacy of 3 mg melatonin/day at bedtime in improving the sleep and alleviating sundowning was shown in 11 elderly AD patients [14] and in 24 patients in other studies [37, 38]. 

Long-term administration of melatonin in the dose of 6-9 mg to 14 AD patients with sleep disorders and sundowning agitation for a period of 2-3 years improved sleep quality [8]. Sundowning, diagnosed clinically in all patients examined was no longer detectable in 12 patients. Another study on 45 AD patients with sleep disturbances, in which 6 mg of melatonin was given daily for 4 months, confirmed sleep improvement and suppression of sundowning [11]. Along with this amelioration, which can already be seen as an important improvement regarding both the patient and the caregiver, the evolution of cognitive alterations in AD patients receiving melatonin seemed to be halted as compared to AD patients not receiving melatonin [8, 9]. The major findings of those open-label studies were confirmed in a double-blind, placebo-controlled study, with regard to sleep-wake rhythmicity, cognitive and non-cognitive functions [3]. 

A large multicenter, randomized, placebo-controlled clinical trial was undertaken to test melatonin efficacy in AD patients [68]. Two dose formulations of oral melatonin were applied and 157 subjects with AD and nighttime sleep disturbance were randomly assigned to one of the following treatment groups: (i) placebo, (ii) 2.5 mg slow-release melatonin, (iii) 10 mg melatonin, given daily for 2 months. In this study melatonin facilitated sleep in a certain number of individuals, but collectively the increase in nocturnal total sleep time and decreased wake after sleep onset, as determined by actigraphy were only apparent as trends in the melatonin-treated groups. On subjective measures, however, caregiver ratings of sleep quality showed significant improvement in the 2.5 mg sustained-release melatonin group relative to placebo [68]. 

Large interindividual differences between patients suffering from a neurodegenerative disease are not uncommon and can explain the erratic results seen with melatonin in fully developed AD. It should be also taken into account that melatonin, though having some sedating and sleep latency-reducing properties, does not primarily act as a sleeping pill, but mainly as a chronobiotic. Since the circadian oscillator system is obviously affected in AD patients showing severe sleep disturbances, the efficacy of melatonin should be expected to depend on disease progression. Indeed, melatonin failed to improve sleep or agitation in two double-blind randomized placebo-controlled trials in institutionalized patients with AD [23, 67]. Thus a major question remains concerning melatonin´s efficacy in advanced AD patients.

Table **1** summarized the published data concerning melatonin treatment of AD patients. Eight reports (5 open-label studies, 2 case reports) (N= 89 patients) supported a possible efficacy of melatonin: sleep quality improved and in patients with AD sundowning was reduced and cognitive decay slowed progression. In 6 double blind, randomized placebo-controlled trials, a total number of 210 AD patients were examined. Sleep was objectively measured by wrist actigraphy (N= 5) and additionally neuropsychological assessment and sleep quality were subjectively evaluated (N= 6). Sleep quality increased and sundowning decreased significantly and cognitive performance improved in 4 studies (N= 143) whereas there was absence of effects in 2 studies (N= 67).

Therefore, the question whether melatonin has a causal value in preventing or treating AD, affecting disease progression of the neuropathology and the driving mechanisms, remains unanswered. Double-blind multicenter studies are needed to further explore and investigate the potential and usefulness of melatonin as an antidementia drug. Its apparent usefulness in symptomatic treatment, concerning sleep, sundowning, etc., even in a progressed state, further underlines the need for such decisive studies.

## MELATONIN AS A THERAPEUTIC AGENT FOR MILD COGNITIVE IMPAIRMENT

As outlined, melatonin acts at different levels relevant to the development and manifestation of AD. The antioxidant, mitochondrial and antiamyloidogenic effects may be seen as a possibility of interfering with the onset of the disease. Therefore, the beginning of treatment is decisive [59]. As seen in Table **1** one cannot expect a profound inhibition of disease progression once a patient is already in an advanced demented state. 

Mild cognitive impairment (MCI) is an etiologically heterogeneous syndrome characterized by cognitive impairment shown by objective measures adjusted for age and education in advance of dementia. Approximately 12% of MCI convert to AD or other dementia disorders every year. Since MCI may represent prodromal AD it should be adequately diagnosed and treated [16]. Indeed, the degenerative process in AD brain starts 20–30 years before the clinical onset of the disease. During this phase, plaques and tangles loads increase and at a certain threshold the first symptom appears. As already mentioned, CSF melatonin levels decrease even in preclinical stages when the patients do not manifest any cognitive impairment (at Braak stages I-II), suggesting that the reduction in CSF melatonin may be an early trigger and marker for AD. Therefore, MCI is the right moment for initiating any melatonin treatment aiming to affect progression of the disease.

The first report on melatonin treatment of 10 MCI patients (6 mg/day for 10 days) indicated that besides enhancing the rest-activity rhythm and improved sleep quality the ability to remember previously learned items improved along with a significant reduction in depressed mood [29]. In another double-blind, placebo-controlled pilot study performed in 26 individuals with age-related MCI, the administration of 1 mg melatonin or placebo at bed time for 4 weeks resulted in improvement of sleep and of scores on the California Verbal Learning Test-interference subtest [54].

In Argentina melatonin was introduced in 1995 as a registered medicament for the treatment of sleep disorders in the elderly, particularly in those whose endogenous melatonin levels are low. Hence, melatonin is often added to the regular treatment of old patients who complain of sleep disorders and memory disturbances in our environment. This gave us the opportunity to carry out a retrospective study of a group of 25 MCI patients who received melatonin (3 – 9 mg per day) for 9 to 18 months in comparison to a similar group of 25 MCI patients who did not receive it [20]. Patients treated with melatonin showed significantly better performance in Mini–Mental State Examination (MMSE) and the cognitive subscale of the Alzheimer’s Disease Assessment Scale (ADAS-Cog). After application of a neuropsychological battery comprising a Mattis´ test, Digit-symbol test, Trail A and B tasks and the Rey´s verbal test, better performance was found in melatonin-treated patients, except for the Digit-symbol test which remained unchanged. Abnormally high Beck Depression Inventory scores decreased in melatonin treated patients, concomitantly with an improvement in wakefulness and sleep quality. The results suggested that melatonin could be a useful add-on drug for treating MCI in a clinic environment [20]. 

A follow up of that study has now been completed and is summarized in Figs. (**1** and **2**). In this expanded retrospective analysis a group of 35 MCI patients received melatonin for 9 to 24 months in comparison to 25 MCI patients who did not receive it. The diagnostic criteria used for MCI were those of Petersen and coworkers [56]. They included amnesic MCI of a degenerative nature (insidious onset and gradual progression), impaired memory, a Clinical Dementia Rating (CDR) < 1.0, a Global Deterioration Scale (GDS) < 2.0, with and a score of 24 to 30 on the MMSE. All participants were informed on the confidentiality of data and on that by no means they would be personally identified in case of publication of the study.

Thirty-five patients in the sample selected had received daily 3 to 9 mg of a fast-release melatonin preparation (Melatol^®^, Elisium S.A., Buenos Aires, Argentina) given p.o. at bedtime. The patients were indicated to take melatonin 30 min before the expected time of sleep every day. Melatonin was given in addition to the individual standard medication prescribed by the attending psychiatrist. The other 25 subjects selected had received the medication prescribed by the attending psychiatrist which did not include melatonin. Patients with a minimum of 9 and a maximum of 24 months of treatment were included. The neuropsychological evaluation included the MMSE, ADAS-Cog and a neuropsychological battery consisting of the Mattis´ test, Digit-symbol test, Trail A and B tasks and the Rey´s verbal test. A 21-item Beck Depression Inventory and a global assessment of wakefulness and sleep quality were also completed for each subject [20]. 

Scores for the MMSE range from 0 to 30, with higher scores indicating better function. Scores for the ADAS-Cog range from 0 to 70, with higher scores indicating poorer function. Mattis´ scores range from 0 to 144 with higher scores indicating better function. Digit-symbol, Trail A and B tasks should be normally completed in less than 10, 120 and 150 seconds, respectively. Normal scores for Rey´s verbal test should be > 85 while those for Beck inventory should be < 20. Scores for wakefulness and sleep quality scales range from 0 to 10, with higher scores indicating better function. In all cases the Δ variation of parameter was computed. Statistical analysis of results was performed by non-parametric Mann-Whitney U tests. 

As shown in Figs. (**1** and **2**), melatonin-treated MCI patients performed better in the MMSE and ADAS-Cog tests, as well in the full neuropsychological battery applied except for the Digit-symbol test. The abnormally high Beck Depression Inventory score decreased in melatonin-treated MCI patients, concomitantly with an improvement in wakefulness and sleep quality. The results confirm and extend the observations supporting a role of melatonin as a useful add-on drug for treating MCI in a clinic environment. 

A randomized controlled trial on the effect of bright light and melatonin on cognitive and noncognitive function in elderly residents of group care facilities was recently published [64]. The authors concluded that light has a benefit in improving some cognitive and noncognitive symptoms of MCI which was amplified by the conjoint administration of melatonin. Melatonin alone had an adverse effect on mood. In other two similar studies, one of them using the prolonged release preparation of melatonin (Circadin^TM^) recently approved by the European Medicines Agency, melatonin resulted in significant and clinically meaningful improvements in sleep quality, morning alertness, sleep onset latency and quality of life in old patients with mild cognitive impairment [22, 80]. In these studies melatonin treatment al improved mood.

Table **2** shows a summary of published data concerning melatonin treatment of MCI patients. Five double blind, randomized placebo-controlled trials and 1 open-label retrospective study (N= 651) all agree in indicating that treatment with daily evening melatonin improves sleep quality and cognitive performance in MCI.

## CONCLUSION

The mechanisms that account for therapeutic effects of melatonin in AD and MCI patients remain to be elucidated. Since the symptomatic actions become relatively rapidly apparent, they should be initially of a chronobiological nature. Melatonin treatment has been shown to promote mainly non-rapid eye movement (REM) sleep in the elderly [47] and is found beneficial in AD by supporting restorative phases of sleep. The chronobiological aspect is underlined by a study on golden hamsters, in which melatonin was able to protect against the circadian changes produced by Aβ_25-35_ microinjection into the SCN [21]. Regardless of the mechanistic details, all pertinent data unanimously direct to a sleep-promoting effect of melatonin in AD and MCI patients, as generally in elderly insomniacs. 

Although there is evidence to postulate melatonin as a useful ad-on therapeutic tool in MCI, larger double-blind multicenter studies are urgently needed to further explore and investigate the potential and usefulness of melatonin as an antidementia drug. Its apparent usefulness in symptomatic treatment, concerning sleep, sundowning or cognitive impairment, even in a progressed state, further underlines the need for such decisive studies.

## Figures and Tables

**Fig. (1):**
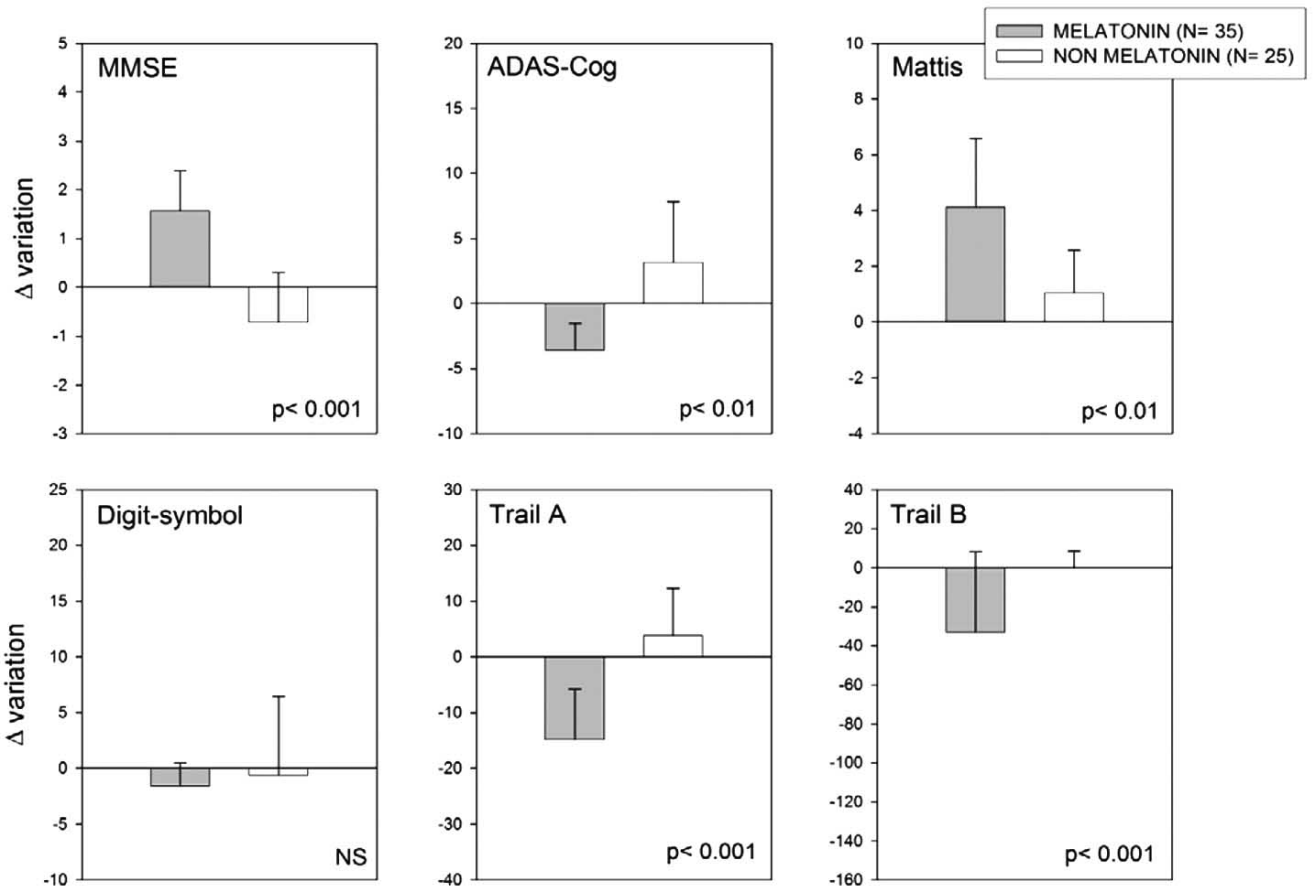
Retrospective analysis of 60 outpatients complaining of MCI symptoms, 35 of which received daily 3 to 9 mg of a fast-release melatonin preparation p.o. at bedtime for 9 to 24 months. Melatonin was given in addition to the individual standard medication prescribed by the attending psychiatrist. The other 25 subjects selected received the medication prescribed by the attending psychiatrist which did not include melatonin. Δ   Variation of neuropsychological evaluation including are depicted. See text for further details. Shown are the means ± SEM. P values denote differences in Z values between final and initial neuropsychological evaluation after a non parametric Mann-Whitney U test.

**Fig. (2):**
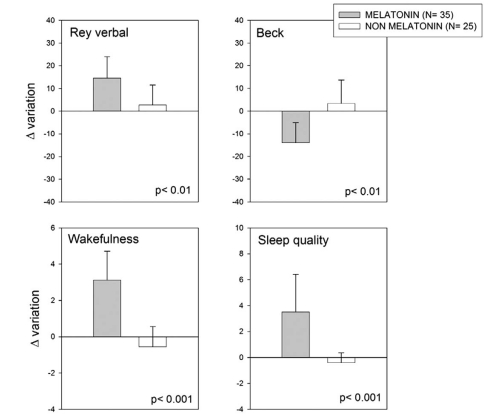
Retrospective analysis of 60 outpatients complaining of MCI symptoms, 35 of which received daily 3 to 9 mg of a fast-release melatonin preparation p.o. at bedtime for 9 to 24 months. For details see Legend to Fig. (1). Shown are the means ± SEM.

**Table 1.** Clinical Studies on Melatonin Efficacy in AD

Design	Subjects (M, F)	Treatment	Study´s Duration	Measured	Results	Reference(s)
Open-label study	10 (6, 4) demented patients	3 mg melatonin p.o. daily at bed time	3 weeks	Daily logs of sleep and wake quality completed by caretakers	Seven out of ten dementia patients having sleep disorders treated with melatonin showed a significant decrease in sundowning and reduced variability of sleep onset time	[17]
Open-label study	14 (8, 14) AD patients	9 mg melatonin p.o. daily at bed time	22 to 35 months	Daily logs of sleep and wake quality completed by caretakers. Neuropsychological assessment.	At the time of assessment, a significant improvement of sleep quality was found. Sundowning was not longer detectable in 12 patients and persisted, although attenuated in 2 patients. Clinically, the patients exhibited lack of progression of the cognitive and behavioral signs of the disease during the time they received melatonin.	[8]
Case report	Monozygotic twins with AD of 8 years duration	One of the patients was treated with melatonin 9 mg p.o. daily at bed time.	36 months	Neuropsychological assessment. Neuroimaging.	Sleep and cognitive function severely impaired in the twin not receiving melatonin as compared to the melatonin-treated twin.	[9]
Open-label, placebo-controlled trial	14 AD patients	6 mg melatonin p.o. daily at bed time or placebo	4 weeks	Daily logs of sleep and wake quality completed by caretakers. Actigraphy	The 7 AD patients receiving melatonin showed a significantly reduced percentage of nighttime activity compared to a placebo group.	[45]
Open-label study	11 (3, 8) AD patients	3 mg melatonin p.o. daily at bed time	3 weeks	Daily logs of sleep and wake quality completed by the nurses.	Analysis revealed a significant decrease in agitated behaviors in all three shifts, and a significant decrease in daytime sleepiness.	[14]
Open-label study	45 (19, 26) AD patients	6–9 mg melatonin p.o. daily at bed time	4 months	Daily logs of sleep and wake quality completed by caretakers. Neuropsychological assessment.	Melatonin improved sleep and suppressed sundowning, an effect seen regardless of the concomitant medication employed to treat cognitive or behavioral signs of AD.	[11]
Randomized double blind placebo controlled cross over study	25 AD patients	6 mg of slow release melatonin p.o. or placebo at bed time	7 weeks	Actigraphy	Melatonin had no effect on median total time asleep, number of awakenings or sleep efficiency.	[67]
Double-blind, placebo-controlled study	20 (3, 17) AD patients	Placebo or 3 mg melatonin p.o. daily at bed time	4 weeks	Actigraphy. Neuropsychological assessment.	Melatonin significantly prolonged the sleep time and decreased activity in the night. Cognitive function was improved by melatonin.	[3]
Randomized, placebo-controlled clinical trial	157 (70, 87) AD patients	2.5-mg slow-release melatonin, or 10-mg melatonin or placebo at bed time	2 months	Actigraphy. Caregiver ratings of sleep quality	Non significant trends for increased nocturnal total sleep time and decreased wake after sleep onset were observed in the melatonin groups relative to placebo. On subjective measures, caregiver ratings of sleep quality showed improvement in the 2.5-mg sustained-release melatonin group relative to placebo.	[68]
Open-label study	7 (4, 3) AD patients	3 mg melatonin p.o. daily at bed time	3 weeks	Actigraphy. Neuropsychological assessment.	Complete remission of day night rhythm disturbances or sundowning was seen in 4 patients, with partial remission in other 2.	[37]
Randomized, placebo-controlled study	17 AD patients	3 mg melatonin p.o. daily at bed time (7 patients). Placebo (10 patients)	2 weeks	Actigraphy. Neuropsychological assessment.	In melatonin-treated group, actigraphic nocturnal activity and agitation showed significant reductions compared to baseline.	[38]
Randomized, placebo-controlled study	50 AD patients	Morning light exposure (2,500 lux, 1 h) and 5 mg melatonin (n= 16) or placebo (n= 17) in the evening. Control subjects (n=17) received usual indoor light (150-200 lux).	10 weeks	Nighttime sleep variables, day sleep time, day activity, day:night sleep ratio, and rest-activity parameters were determined using actigraphy.	Light treatment alone did not improve nighttime sleep, daytime wake, or rest-activity rhythm. Light treatment plus melatonin increased daytime wake time and activity levels and strengthened the rest-activity rhythm.	[15]
Case report	68-year-old man with AD who developed rapid eye movement (REM) sleep behavior disorder	5 – 10 mg melatonin p.o. daily at bed time.	20 months	Polysomnography	Melatonin was effective to suppress REM sleep behavior disorder	[2]
Randomized, placebo-controlled study	41 (13, 28) AD patients	Melatonin (8.5 mg immediate release and 1.5 mg sustained release) (N = 24) or placebo (N = 17) administered at 10:00 P.M.	10 days	Actigraphy.	There were no significant effects of melatonin, compared with placebo, on sleep, circadian rhythms, or agitation.	[23]

**Table 2.** Clinical Studies on Melatonin Efficacy in MCI

Design	Subjects (M, F)	Treatment	Study´s Duration	Measured	Results	Reference(s)
Double-blind, placebo-controlled,crossover study	10 (4, 6) patients with MCl	6 mg melatonin p.o. daily at bed time	10 days	Actigraphy. Neuropsychological assessment.	Melatonin enhanced the rest-activity rhythm and improved sleep quality (reduced sleep onset latency and number of transitions from sleep to wakefulness). Total sleep time unaffected. The ability to remember previously learned items improved along with a significant reduction in depressed mood.	[29]
Double-blind, placebo-controlled pilot study	26 individuals with age-related MCI	1mg melatonin p.o. or placebo at bed time	4 weeks	Sleep questionnaire and a battery of cognitive tests at baseline and at 4 weeks	Melatonin administration improved reported morning "restedness" and sleep latency after nocturnal awakening, and also improved scores on the California Verbal Learning Test-interference subtest.	[54]
Open-label, retrospective study	50 (13, 37) MCI outpatients	25 had received daily 3-9 mg of a fast-release melatonin preparation p.o. at bedtime. Melatonin was given in addition to the standard medication	9-18 months	Daily logs of sleep and wake quality. Initial and final neuropsychological assessment.	Patients treated with melatonin showed significantly better performance in neuropsychological assessment. Abnormally high Beck Depression Inventory scores decreased in melatonin-treated patients, concomitantly with an improvement in wakefulness and sleep quality.	[20]
Randomized, double blind, placebo-controlled study	354 individuals with age-related cognitive decay	prolonged release melatonin (Circadin, 2 mg) or placebo, 2 h before bedtime	3 weeks	Leeds Sleep Evaluation and Pittsburgh Sleep Questionnaires, Clinical Global Improvement scale score and quality of life.	Melatonin treatment resulted in significant and clinically meaningful improvements in sleep quality, morning alertness, sleep onset latency and quality of life	[80]
Long-term, double-blind, placebo-controlled, 2 x 2 factorial randomized study	189 (19, 170) individuals with age-related cognitive decay	Long-term daily treatment with whole-day bright (1000 lux) or dim (300 lux) light. Evenin*g *melatonin (2.5 mg) or placebo administration	1 to 3.5 years	Standardized scales for cognitive and noncognitive symptoms, limitations of activities of daily living, and adverse effects assessed every 6 months.	Light attenuated cognitive deterioration and also ameliorated depressive symptoms. Melatonin shortened sleep onset latency and increased sleep duration but adversely affected scores for depression. The combined treatment of bright light plus melatonin showed the best effects.	[64]
Prospective, randomized, double-blind, placebo-controlled, study	22 (15, 7) individuals with age-related cognitive decay	Participants received 2 months of melatonin (5 mg p.o. /day) and 2 months of placebo	2 months	Sleep disorders were evaluated with the Northside Hospital Sleep Medicine Institute (NHSMI) test. Behavioral disorders were evaluated with the Yesavage Geriatric Depression Scale and Goldberg Anxiety Scale.	Melatonin treatment significantly improved sleep quality scores. Depression also improved significantly after melatonin administration.	[22]
